# Effects of Ramadan fasting on glucose homeostasis and adiponectin levels in healthy adult males

**DOI:** 10.1186/s40200-015-0183-9

**Published:** 2015-07-07

**Authors:** Justin V. Gnanou, Brinnell A. Caszo, Khalifah M. Khalil, Shahidah L. Abdullah, Victor F. Knight, Mohd Z. Bidin

**Affiliations:** Faculty of Medicine and Defence Health, National Defence University of Malaysia, Kem Sungai Besi, Kuala Lumpur, 57000 Malaysia

**Keywords:** Ramadan, Intermittent fasting, Insulin sensitivity, Glucose homeostasis, Malaysian, Adiponectin

## Abstract

**Background:**

Adiponectin is a hormone secreted by adipocytes during the fasting phase of the fast-fed cycle. Ramadan fasting involves prolonged fasting for up to twelve hours and thus could lead to increased secretion of adiponectin by adipocytes. However, studies on the role of adiponectin on glucose and body weight homeostasis during Ramadan fasting is still a matter of controversy. Thus the specific aim of this study was to assess the effect of fasting during Ramadan on the adiponectin levels, body weight and glucose homeostasis in healthy male Malaysian subjects.

**Methods:**

Twenty healthy male (19–23 years) Muslim subjects were followed up during the fasting month of Ramadan. Anthropometry and blood samples were taken one week before and during the fourth week of fasting. Plasma glucose, insulin and adiponectin were estimated and insulin sensitivity indices were estimated using the Homeostasis Model Assessment.

**Results:**

Subjects experienced a significant decrease in body weight (2.4 %, *p* < 0.001) and body mass index (5.5 %, *p* < 0.01). There was also a significant decrease of 12.3 %, 52.8 % and 45.6 % of plasma glucose, insulin and adiponectin respectively (*p* < 0.01). The drop in adiponectin was positively correlated with the decrease in body weight (r = 0.45, *p* < 0.05). There was also a significant increase in insulin sensitivity and a decrease in insulin resistance (*p* < 0.01).

**Conclusions:**

These results indicate that Ramadan fasting in young healthy individuals has a positive impact on the maintenance of glucose homeostasis. It also shows that adiponectin levels dropped along with significant loss in weight. We feel caloric restriction during the Ramadan fasting is in itself sufficient to improve insulin sensitivity in healthy individuals.

## Background

Fasting is a behavioral practice involving abstention from or restriction of food, water or certain select nutrient groups. It is widely practiced by followers of a number of religions [1]. Some of the best studied models of this activity include the Ramadan fasting month among the followers of Islam. Fasting during the Ramadan month is a chronic diurnal fast lasting for a period of 30 days. This fasting process provides a unique metabolic model that includes abstinence from food and water from dawn to dusk (12 h) as well as a reduction in the meal frequency [2]. At the end of the daily fasting period, the fast is broken by consumption of food during the night time [3]. However, the type and amount of food consumed during this breaking of fast differs culturally as well as due to the economic status of the community or the country. Studies on body weight gain/loss after the 30 days of fasting have shown that depending on the economic situation of the country/society, individuals actually gain weight in economically well placed countries [4, 5], while individuals lose weight in economically deprived countries [6, 7].

Adipokines are hormones secreted by the adipose tissue that regulate and maintain energy and body weight homeostasis. Adiponectin is an adipokine whose secretion is increased during the fasting or a phase of under nutrition. The main function of adiponectin is to promote fat oxidation and improve sensitivity by increasing glucose uptake in skeletal muscles [8]. These actions are mediated by AdipoR1 (Adiponectin Receptor 1) and AdipoR2 (Adiponectin Receptor 2) receptors and via 5′ AMP-activated protein kinase (AMPK) activation in these tissues [9, 10]. Studies on the role of adiponectin during Ramadan fasting or intermittent fasting with caloric restriction have shown conflicting results. In a study done on healthy subjects in Saudi Arabia, a significant reduction in adiponectin levels was observed with no change in body mass or body fat after two weeks of Ramadan fasting [11]. However, another study among healthy Pakistani male subjects, observed a 50 % increase in adiponectin levels during Ramadan fasting [12]. Thus the coordinated secretion of adiponectin from the adipocytes might be affected during intermittent fasting during Ramadan depending on the type and amount of food consumed.

Therefore, this study was designed to assess the effect of intermittent fasting during Ramadan on the adiponectin levels and its relationship with body weight and insulin sensitivity in healthy male Malaysian subjects.

## Subjects and methods

Subjects were recruited by advertisment from the National Defence University of Malaysia from among students of Year one medical program from a batch of 62 students. Male subjects, above the age of 18, who volunteered and gave informed consent, did not have any significant past medical history, were nonsmokers and who were observing the Ramadan fast were included in the study. The study protocol was explained to these students in detail and informed consent was obtained. The students were regularly motivated to continue with the study however during the course of the study, five students dropped out due to personal reasons. The study protocol was approved by the institutional ethical review board of National Defence University of Malaysia.

The study protocol consisted of two phases – phase one or pre-Ramadan phase and phase two; in the fourth week of Ramadan. Phase one was conducted in the week prior to the beginning of the Ramadan fast. In order to maintain the same duration of fasting (eight hours) before collection of blood samples in both phase one and two, blood samples were collected at one pm. During phase 1, subjects were asked to complete their morning meal by 5 am and fast until 1 pm. In this manner, both phase one and two samples were collected 8 h after the morning meal.

On the day of the experiment (phase one – pre-Ramadan), body weight and height (Seca GmBH & Co Kg, Hamburg, Germany) was measured by standard techniques and expressed in kilograms and meters respectively [13]. Body weight was measured with minimal clothing and without shoes using a digital scale with a 100-g maximum error, while the height was measured in subjects in the standing position without shoes, with five points of the body touching the wall in centimeters (cm) to the nearest 0.1 cm. Mid upper arm circumference was measured at the midpoint between the tip of the shoulder and the tip of the elbow of the non-dominant arm, waist circumference was measured at the level of the umbilicus, and hip circumference was measured at the trochanter levels and was expressed in centimeters. Body mass index (BMI) using the formula body weight (kilograms) divided by height (meters) squared and waist-to-hip ratio (WHR) was calculated from these measurements. All anthropometric measurements were made by the same observer, as previously described [13]. Blood and urine samples were collected using standard aseptic precautions. In order to ascertain the fasting status, fasting glucose was measured immediately. The blood samples were transferred into appropriate containers, centrifuged and the separated serum/plasma was stored at – 80 °C until analysis.

The second phase of the experiment was carried out during the fourth week of Ramadan month. Eight hours after the beginning of the fast (at 1.00 pm), a protocol similar to that of phase one was carried out. This ensured there was no difference in the duration of fasting in both phases in order to avoid daily variations in the insulin and adiponectin secretion.

The biochemical analysis of the blood samples were carried out at the Multipurpose Research Laboratory of the Faculty of Medicine, National Defence University of Malaysia. Glucose was measured immediately after blood collection using the glucose hexokinase method. Hormones, insulin and adiponectin were measured using commercially available enzyme immunoassay kits as follows: Insulin (Mercodia AB, Sylveniusgatan 8A, Uppsala, Sweden; sensitivity, 1 mU/L; intra-assay coefficients of variation, 2.8 – 4.0 %); Adiponectin (eBioscience, Vienna Biocenter, Austria; sensitivity, 0.01 ng/ml; intra-assay coefficients of variation, 1.9 – 6.3 %). Urine and serum osmolality were measured using freezing point depression (Advanced 3250 Osmometer, Advanced Instruments Inc., Norwood, MA, USA). All data were expressed as means ± standard deviation. Insulin sensitivity and insulin resistance were estimated using the homeostasis model (HOMA) [14] from basal plasma glucose and plasma insulin values. Normality of data was checked using the Kolmogorov-Smirnov test. Student’s *t*-test was used to determine the effect of four-weeks of intermittent fasting on all parameters measured before and after fasting. Changes between the values after four weeks of fasting and the pre-Ramadan value were calculated for all parameters and Pearson’s correlation analysis was used to study their association. Statistical significance was accepted at *p* < 0.05.

## Result

**Fig. 1:**
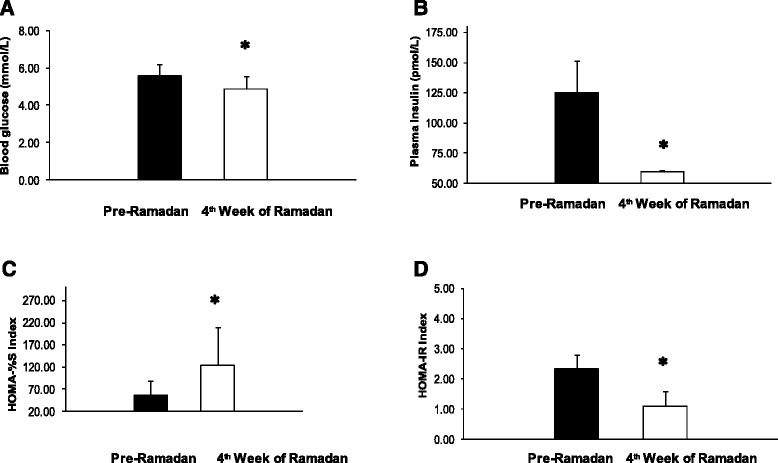
Changes in blood glucose, plasma insulin and HOMA indices after four weeks of Ramadan fasting. **a** = Fasting Blood Glucose; **b** = Fasting Plasma Insulin. **c** = Insulin Sensitivity HOMA-%B; **d** = Insulin Resistance HOMA IR; Data represented as mean ± standard deviation. *: *p* < 0.01

**Fig. 2:**
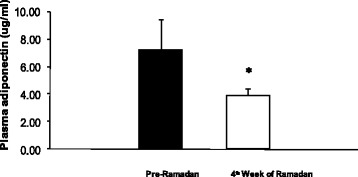
Effect of Ramadan fasting on plasma adiponectin. Data represented as mean ± standard deviation. *: *p* < 0.01

**Fig. 3:**
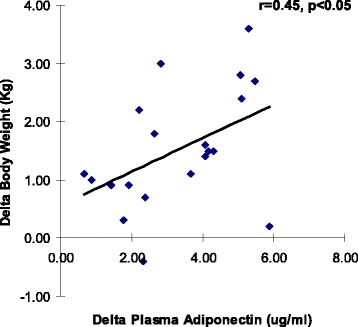
Correlation between changes in plasma adiponectin and body weight during the four week fasting period. A significant correlation (r = 0.45, *p* < 0.05) was obtained showing an association between changes in plasma adiponectin and body weight after four weeks of fasting

Four weeks after observing the Ramadan fast, a significant reduction (2.4 %) in body weight (*p* < 0.01) and also a significant reduction (5.5 %) in BMI (*p* < 0.01) were observed. There was also a significant reduction (3.2 %) in mid upper arm circumference (*p* < 0.01), however no difference in waist to hip ratio was observed (Table 1). In order to study the effect of Ramadan fasting on glucose homeostasis, plasma glucose and plasma insulin were measured. As shown in Fig. 1, there was a significant decrease in both the parameters (*p* < 0.01). There was also a significant increase in insulin sensitivity and a significant decrease in insulin resistance (*p* < 0.01). Finally, in order to study the role of adiponectin in intermittent fasting, plasma adiponectin was measured. We found a significant reduction in plasma adiponectin (Fig. 2) (*p* < 0.01). Findings from linear regression analysis revealed a significant positive correlation (r = 0.45, *p* < 0.05) between the change in plasma adiponectin and change in body weight due to the intermittent fasting (Fig. 3).

**Table 1:** Effect of Ramadan fasting on the anthropometric parameters

Anthropometric parameters	Pre-Ramadan	4^th^ week of Ramadan	% reduction	p value
Body weight (kg)	63.07 ± 8.19	61.55 ± 8.11	2.4	<0.01
Body Mass Index (kg/m^2^)	22.23 ± 2.24	21.23 ± 2.36	5.5	<0.01
Upper Mid Arm Circumference (cm)	27.73 ± 3.17	26.85 ± 2.81	3.2	<0.01
Waist to Hip Ratio	0.84 ± 0.03	0.84 ± 0.05	-	NS

## Discussion

This study was designed to determine the effect of intermittent fasting that is practiced by Muslims during the month of Ramadan. Our subjects were healthy male subjects in their early twenties. They were university students who maintained normal physical activity status during the fasting month. We found a significant decrease in body weight as well as BMI in these subjects after the four weeks of intermittent fasting. On average the subjects lost 1.52 kg during this period. Dehydration could be a cause for this reduction in body weight [15]. Studies have shown that the intermittent dehydration observed during Ramadan could lead to an overall reduction in total body water [16] and hence body weight. Thus to rule out dehydration as the cause of weight loss, we measured urine and plasma osmolality and later calculated urine/plasma osmolality ratio – a measure of hydration status of an individual [17]. We observed no significant difference in urine/plasma osmolality ratio between the two phases. Thus subjects were able to maintain similar hydration status during both phases of the study. This also ruled out the factor of hydration affecting the variations in blood parameters between the two phases. Weight loss may be primarily attributed to caloric restriction as part of the intermittent fasting. This finding of weight loss has been consistent with previous studies on subjects from a similar age group [6, 18, 19]. However few studies have found no change in body weight after Ramadan fasting [20–23], while Frost and Pirani [24] showed an increase in body weight. These differences might be due to differences in physical activity of subjects and the type and amount of food consumed during the non-fasting period of the fast [25]. Subjects resided in hostels within the university campus and participated in the same regular physical activity training schedule as part of their curriculum. Though the physical activity was less strenuous during the fasting month, subjects did participate in daily training. Also since all our subjects had meals (during the breaking of the fast) at the same venue (hostel mess) during the study, the meals were not excessive and were limited in quantity. Thus subjects would not be able to over eat while breaking the fast. These factors could have played a role in the loss of body weight observed in our study. Our study also found a significant decrease in mid upper arm circumference after Ramadan fasting. Mid upper arm circumference is considered as a good measure of acute changes in nutritional status in adults and recently it has been accepted as a simpler substitute to BMI [26]. Thus, decrease in mid upper arm circumference corroborates weight loss and decreased BMI seen in our study.

Loss of body weight due to caloric restriction [27], intermittent fasting [28] or exercise [29] has been shown to increase insulin sensitivity and increase glucose uptake into peripheral tissues. Our findings showed a significant reduction in fasting plasma glucose levels. Though few studies have shown no change [30] or even increase [31] in glucose levels after Ramadan fasting, our study findings are in line with other studies that have shown similar decrease in plasma glucose levels [32, 33]. The difference in findings between studies on plasma glucose levels after Ramadan fasting could be attributed to the individual variation in glycogen storage, physical activity and dietary habits. We found a decrease in fasting plasma insulin levels, similar to the decrease seen in plasma glucose levels. This may be explained by the fact that since there is less plasma glucose to be taken up by the peripheral tissue, hence the need for lower insulin in the plasma. This might explain the increase in insulin sensitivity among our subjects. Insulin sensitivity has been shown to be increased after Ramadan fasting among patients with metabolic syndrome [34] and in patients with type two diabetes mellitus [35]. Heilbronn et al. found an increase in insulin sensitivity after three weeks of alternative fasting among healthy subjects [36]. However a study on healthy subjects with uninterrupted fasting for 72-h, showed a significant worsening of insulin sensitivity. Thus, fasting of longer duration seems to have a beneficial effect on glucose homeostasis by increasing the insulin sensitivity. Interestingly it has been shown that weight loss (after a weight loss program) causes a reduction in fatty acid mobilization (30 %) and an improvement of 60 % of insulin sensitivity, independent of the effect of exercise [37]. Thus insulin sensitivity may be the effect of body weight loss rather the cause of it. However changes in insulin sensitivity showed no correlation with changes in body weight in our study.

It has been proposed that adiponectin acts a ‘spacer’ between small adipocytes and thus maintains good perfusion of adipocytes by interstitial fluid. This helps adipocytes remain metabolically active [38]. Besides this, adiponectin improves insulin sensitivity by increasing expression of insulin receptors (IRS-2) by activation of signal transducer and activator of transcription-3 (STAT3). Though we found an improvement in insulin sensitivity at the end of Ramadan fasting, plasma adiponectin levels dropped significantly. Very few studies have estimated adiponectin levels during the Ramadan fasting with contrasting findings of either no change [12] or decrease in its level [11]. It may be that as fasting progressed, the amount of adiponectin required for the maintenance of its various functions had also decreased, hence the decrease in its plasma level. The data also showed a significant positive correlation between the change in body weight and the change in adiponectin levels over the duration of the study. Thus as body weight decreased during the fasting period, adiponectin levels also dropped showing that in healthy adult subjects, adiponectin levels may be directly related to body weight changes.

This study has limitations in that, we did not quantitate food intake and physical activity in our subjects. The type, the caloric value of our subjects’ diet would have added more valuable data to our study. However all subjects resided in the hostel, took their meals in the same mess and participated in the same training routines. Also, an assessment of body composition would enable us to tease out the relationship between adiponectin and body fat mass.

## Conclusion

In conclusion, intermittent fasting as practiced during Ramadan by healthy subjects has a beneficial effect on metabolism. Though the subjects were not put on any dietary restriction during the Ramadan fasting month, we observed a decrease in body weight and BMI, an improvement in insulin sensitivity (with decrease in both fasting glucose and insulin) as well as decrease in plasma adiponectin levels. Further studies are required to elucidate the mechanism of improved insulin sensitivity and fall in adiponectin levels in the healthy adult subjects.

## Abbreviations

AdipoR1: Adiponectin Receptor 1
AdipoR2: Adiponectin Receptor 2
AMPK: 5′ adenosine monophosphate-activated protein kinase
BMI: Body mass index
HOMA: Homeostasis model assessment
IRS-2: Insulin receptor substrate −2
Kg: Kilogram
STAT3: Signal transducer and activator of transcription 3
VLDL: Very low density lipoprotein
WHR: Waist to hip ratio

## References

[1] Trepanowski JF, Bloomer RJ (2010). The impact of religious fasting on human health. Nutr J.

[2] Ibrahim WH, Habib HM, Jarrar AH, Al Baz SA (2008). Effect of Ramadan fasting on markers of oxidative stress and serum biochemical markers of cellular damage in healthy subjects. Ann Nutr Metab.

[3] Aksungar FB, Eren A, Ure S, Teskin O, Ates G (2005). Effects of intermittent fasting on serum lipid levels, coagulation status and plasma homocysteine levels. Ann Nutr Metab.

[4] Hajek P, Myers K, Dhanji AR, West O, McRobbie H (2012). Weight change during and after Ramadan fasting. J Public Health (Oxf).

[5] Bakhotmah BA (2011). The puzzle of self-reported weight gain in a month of fasting (Ramadan) among a cohort of Saudi families in Jeddah, Western Saudi Arabia. Nutr J.

[6] Ziaee V, Razaei M, Ahmadinejad Z, Shaikh H, Yousefi R, Yarmohammadi L (2006). The changes of metabolic profile and weight during Ramadan fasting. Sing Med J.

[7] Salahuddin M, Sayed Ashfak AH, Syed SR, Badaam KM. Effect of Ramadan Fasting on Body Weight, (BP) and Biochemical Parameters in Middle Aged Hypertensive Subjects: An Observational Trial. J Clin Diagn Res. 2014;8(3):16–18. 10.7860/JCDR/2014/8108.4092 PMC4003623 24783068

[8] Lee B, Sha J (2012). Adiponectin and lipid metabolism in skeletal muscle. Acta Pharm Sin B.

[9] Yamauchi T, Kamon J, Ito Y, Tsuchida A, Yokomizo T, Kita S (2003). Cloning of adiponectin receptors that mediate antidiabetic metabolic effects. Nature.

[10] Mao X, Kikani CK, Riojas RA, Langlais P, Wang L, Ramos FJ (2006). APPL1 binds to adiponectin receptors and mediates adiponectin signaling and function. Nat Cell Biol.

[11] Ajabnoor GM, Bahijri S, Borai A, Abdulkhaliq AA, Al-Aama JY, Chrousos GP (2014). Health Impact of Fasting in Saudi Arabia during Ramadan: Association with Disturbed Circadian Rhythm and Metabolic and Sleeping Patterns. PLoS One.

[12] Mesci B, Oguz A, Erok B, Coksert Kilic D, Akalin A (2012). Effect of intended fasting on Serum Leptin, Adiponectin and Ghrelin levels. Pak J Med Sci.

[13] WHO Expert Committee (1995). Physical status: the use and interpretation of anthropometry. Report of a WHO Expert Committee.

[14] Matthews DR, Hosker JP, Rudenski AS, Naylor BA, Treacher DF, Turner RC (1985). Homeostasis model assessment: insulin resistance and beta-cell function from fasting plasma glucose and insulin concentrations in man. Diabetologia.

[15] Thomas DR (2002). Distinguishing starvation from cachexia. Clin Geriatr Med.

[16] Leiper JB, Prastowo SM (2000). Effect of fasting during Ramadan on water turnover in men living in the tropics. J Physiol.

[17] Cheuvront SN, Kenefick RW, Charkoudian N, Sawka MN (2013). Physiologic basis for understanding quantitative dehydration assessment. Am J Clin Nutr.

[18] Fedail SS, Murphy D, Salih SY, Bolton CH, Harvey RF (1982). Changes in certain blood constituents during Ramadan. Am J Clin Nutr.

[19] Rahman MM, Rashid S, Basher S, Sultana S, Nomani MZ (2004). Improved serum HDL cholesterol profile among Bangladeshi male students during Ramadan fasting. East Mediterr Health J.

[20] Finch GM, Day JEL, Razak, Welch DA, Rogers PJ (1998). Appetite changes under free- living conditions during Ramadan fasting. Appetite.

[21] el Ati J, Beji C, Danguir J (1995). Increased fat oxidation during Ramadan fasting in healthy women: an adaptative mechanism for body-weight maintenance. Am J Clin Nutr.

[22] Husain R, Duncan MT, Cheah SH, Ch’ng SL (1987). Effects of fasting in Ramadan on Tropical Asiatic Moslems. Br J Nutr.

[23] Yucel A, Degirmenci B, Acar M, Albayrak R, Haktanir A (2004). The effect of fasting month of Ramadan on the abdominal fat distribution: assessment by computed tomography. Tohoku J Exp Med.

[24] Frost G, Pirani S (1987). Meal frequency and nutritional intake during Ramadan: a pilot study. Hum Nutr Appl Nutr.

[25] Rouhani M, Azadbakht L (2014). Is Ramadan fasting related to health outcomes? A review on the related evidence. J Res Med Sci.

[26] Powell-Tuck J, Hennessy EM (2003). A Comparison of mid upper arm circumference, body mass index and weight loss as indices of undernutrition in acutely hospitalised patients. Clin Nutr.

[27] Weiss EP, Albert SG, Reeds DN, Kress KS, Ezekiel UR, McDaniel JL (2014). Weight Loss-Independent Effect of Calorie Restriction on Insulin Sensitivity and Postprandial Incretin Hormones. J Acad Nutr Diet.

[28] Dubé JJ, Fleishman K, Rousson V, Goodpaster BH, Amati F (2012). Exercise Dose and Insulin Sensitivity: Relevance for Diabetes Prevention. Med Sci Sports Exerc.

[29] Mendelson M, Michallet AS, Monneret D, Perrin C, Estève F, Lombard PR, et al. Impact of exercise training without caloric restriction on inflammation, insulin resistance and visceral fat mass in obese adolescents. Pediatr Obes 2014, doi:10.1111. 10.1111/ijpo.255 25088157

[30] Unalacak M, Kara IH, Baltaci D, Erdem O, Bucaktepe PG (2011). Effects of Ramadan fasting on biochemical and hematological parameters and cytokines in healthy and obese individuals. Metab Syndr Relat Disord.

[31] Nematy M, Alinezhad-Namaghi M, Rashed MM, Mozhdehifard M, Sajjadi SS, Akhlaghi S (2012). Effects of Ramadan fasting on cardiovascular risk factors: a prospective observational study. Nutr J.

[32] Faris MA, Hussein RN, Al-Kurd RA, Al-Fararjeh MA, Bustanji YK, Mohammad MK (2012). Impact of Ramadan intermittent fasting on oxidative stress measured by urinary 15-f(2 t)-isoprostane. J Nutr Metab.

[33] Larijani B, Zahedi F, Sanjari M, Amini MR, Jalili RB, Adibi H (2003). The Effect of Ramadan Fasting on Fasting Serum Glucose in Healthy Adults. Med J Malaysia.

[34] Shariatpanahi ZV, Shariatpanahi MV, Shahbazi S, Hossaini A, Abadi A (2008). Effect of Ramadan fasting on some indices of insulin resistance and components of the metabolic syndrome in healthy male adults. Br J Nutr.

[35] Yarahmadi SH, Larijani B, Bastanhagh MH, Pajouhi M, Baradar Jalili R, Zahedi F (2003). Metabolic and clinical effects of Ramadan fasting in patients with type II diabetes. J Col Physicians Surg Pa.

[36] Heilbronn LK, Civitarese AE, Bogacka I, Smith SR, Hulver M, Ravussin E (2005). Glucose Tolerance and Skeletal Muscle Gene Expression in Response to Alternate Day Fasting. Obes Res.

[37] Schenk S, Harber MP, Shrivastava CR, Burant CF, Horowitz JF (2009). Improved insulin sensitivity after weight loss and exercise training is mediated by a reduction in plasma fatty acid mobilization, not enhanced oxidative capacity. J Physiol.

[38] Nakamura MT, Yudell BE, Loor JJ (2014). Regulation of energy metabolism by long- chain fatty acids. Prog Lipid Res.

